# Enzymatic synthesis of epothilone A glycosides

**DOI:** 10.1186/s13568-014-0031-1

**Published:** 2014-03-20

**Authors:** Prakash Parajuli, Ramesh Prasad Pandey, Niranjan Koirala, Yeo Joon Yoon, Byung-Gee Kim, Jae Kyung Sohng

**Affiliations:** 1 Institute of Biomolecule Reconstruction (iBR), Department of Pharmaceutical Engineering, SunMoon University, Chungnam 336-708, Asan-si, Republic of Korea; 2 Department of Chemistry and Nano Science, Ewha Womans University, Seoul 120-750, Republic of Korea; 3 Laboratory of Molecular Biotechnology and Biomaterials, School of Chemical and Biological Engineering, Seoul National University, Seoul, Republic of Korea

**Keywords:** Epothilone A glycosides, Glycosylation, Bacillus licheniformis, Chemotherapeutic agent

## Abstract

Epothilones are extremely cytotoxic chemotherapeutic agents with epoxide, thiazole, and ketone groups that share equipotent kinetic similarity with taxol. The in vitro glycosylation catalyzed by uridine diphosphate glucosyltransferase (YjiC) from *Bacillus licheniformis* generated six novel epothilone A glycoside analouges including epothilone A 7-*O*-*β*-D-glucoside, epothilone A 7-*O*-*β*-D-galactoside, epothilone A 3,7-*O*-*β*-D-digalactoside, epothilone A 7-*O*-*β*-D-2-deoxyglucoside, epothilone A 7-*O*-*β*-L-rhamnoside, and epothilone A 7-*O*-*β*-L-fucoside. Epothilone A 7-*O*-*β*-D-glucoside was structurally elucidated by ultra-high performance liquid chromatography-photo diode array (UPLC-PDA) conjugated with high resolution quantitative time-of-flight-electrospray ionization mass spectroscopy (HR-QTOF ESI-MS/MS) supported by one-and two-dimensional nuclear magnetic resonance studies whereas other epothilone A glycosides were characterized by UPLC-PDA and HR-QTOF ESI-MS/MS analyses. The time dependent conversion study of epothilone A to epothilone A 7-*O*-*β*-D-glucoside found to be maximum (~26%) between 3 h to 5 h incubation.

## Introduction

**Figure 1:**
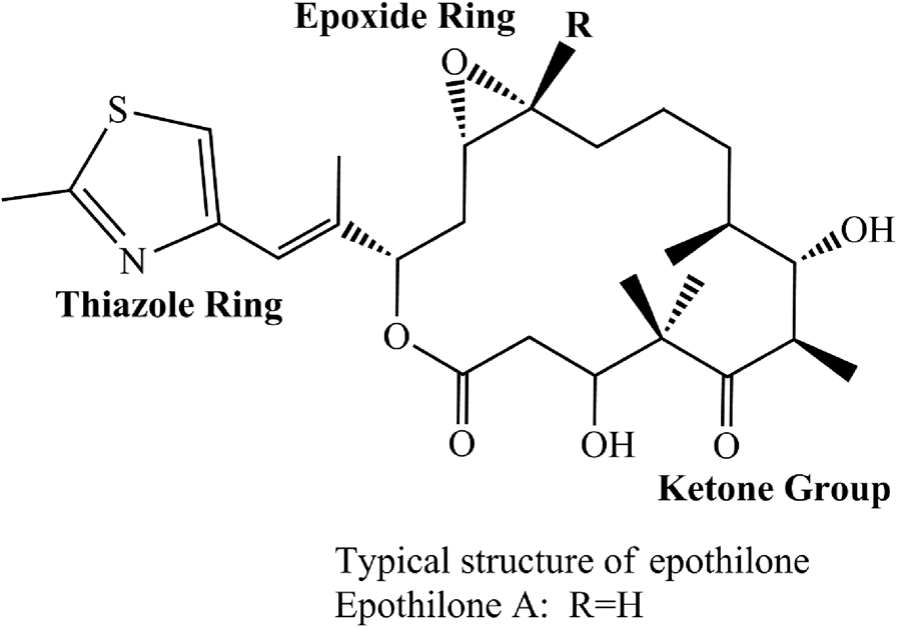
**Typical structure of epothilone.** The structure displays thiazole ring, ketone group and an epoxide ring in epothilone group of compounds.

Epothilones are a class of polypeptide macrolides produced by a few strains of myxo-bacterium such as *Sorangium cellulosum* (Cheng et al. [2008]). Epothilones are structurally characterized as macrolactones with epoxy and keto-groups in a lactone ring and a side chain with a thiazole ring (Figure 1). Epothilones were discovered in 1987 as antifungal agents (Hofle and Reichenbach [2005]). Different analogs of epothione (A–H) containing 29 variants have been reported in *S. cellulosum* but epothilone A and B are the major products with potential applications in therapy and cytotoxic effect in tumor cell lines (Hardt et al. [2001]). Similarly, 37 natural epothilone variants and related compounds were later isolated and confirmed from the same strain by Hofle and Reichenbach in 2001.

Eothilones have been recognized as chemotherapeutic agents against different tumor cell lines including those affected by taxanes with manageable toxicity profiles demonstrated in both preclinical and clinical trials (Lee et al. [2001]; Thomas et al. [2007]). Eothilones are potent inducers of a microtubule stabilizing agent, which binds to β-tubulin during mitotic cell division, resulting in apoptosis or programmed cell death (Lee et al. [2007]). Although paclitaxel (taxol) appears to be the most effective antineoplastic agent in the last decade, but it has several drawbacks such as poor solubility and the use of cremophor (solubilizing agent, causes side effects via hyperlipidemia and abnormal lipoprotein patterns) (Rowinsky et al. [1993]) which leads in search of novel antibiotics. This hurdle has been cleared by introducing epothilones with higher potency against tumor cells including more water soluble properties, and can be administered without any additives compare to paclitaxel (Bollag et al. [1995]). Furthermore, the in vivo cytotoxic activity of epothilone A and B compared with that of paclitaxel against various multi-drug resistances cancer cell lines has also been reported (Carlomagno et al. [2003]).

Natural products have long been a source of cancer therapeutic agents. Therefore, it is a continual need of anticancer drug discovery and their modifications either structurally or functionally to enhance the therapeutic applications (Padilla and Furlan [2009]). Enzymatic synthesis and modification of such natural products is a current need to significantly enhance their pharmacological properties and biological actions (Luzhetskyy and Bechthold [2008]; Thibodeaux et al. [2008]). The majority of natural products have carbohydrate attached, that helps in cell recognition with enhance bioavailability including maintenance of cell integrity, molecular recognition, pathogen virulence, and molecular defense mechanism (Song et al. [2013]; Singh et al. [2012]). Glycosylation as a post-modification process is an effective tool to diversify natural products (Simkhada et al. [2010]). Glycosylation broadens the biological potency and applications of compounds by altering physical, chemical, and biological properties (Singh et al. [2012]). For example, paclitaxel i.e. 7-β-xylosyl-10 deacetyltaxol, a glycoconjugate of the taxane prodrug, has more than two orders of magnitude improved water solubility without a reduction in clinical efficacy. The similar property was shown with water solubility of geldanamycin analogs (Wu et al. [2012]; Cheng et al. [2013]) as the clinical utility of geldanamycin has been compromised, although it is a potent anticancer drug due to its poor water solubility and severe toxic effects (Wu et al. [2012]). Many such hurdles have been overcome through the use of glycosylated analogues which are difficult to obtain from the parent compound (Lomino et al. [2013]). Most natural products currently used as therapeutics derive their biological functions from the sugar components present in their structure; however, alterations could broaden their pharmacological properties (Chang et al. [2011]). For example, the DNA binding affinity and cytotoxicity of rebeccamycin (an antitumor drug with potential topoisomerase I poisoning effects) can be altered by variation in sugar moieties (Animati et al. [2008]). Doxorubicin (similar antitumor agent with strong chemotherapeutic applications) has no antitumor activity if the sugar moiety is removed (Han et al. [2011]); and tylosin and erythromycin (antibiotics) have sugar moieties that may affect their molecular mechanism of action (Langenhan et al. [2005]). Amphotericin B (an antifungal agent) has a glycoside attached that enhances its solubility and pharmacokinetics (Elgart et al. [2010]).

Glycosylation is defined as coupling of a sugar moiety with an aglycone acceptor, which can be achieved by enzymatic, chemical synthesis, and chemoenzymatic approaches (neo-glycosylation). The first approach is catalyzed by enzymes called UDP-glycosyltransferases, which belong to the GT1 family proteins in the CAZy classification (http://www.cazy.org/). Carbohydrate moieties are usually attached to molecules via the *O-*glycosidic bond but some are linked through *C–* or *N-*glycosidic linkages (Salas and Mendez [2007]). The number and type of conjugated sugar units on the compound depends on the enzyme used for catalysis.

Here, we generated epothilone A glycoside analogues by applying a YjiC from *Bacillus licheniformis* DSM 13. YjiC conjugates different sugar units in plant natural products with low molecular weight, including chalcone (Pandey et al. [2013a]), flavonols (Pandey et al. [2013b]) and potential anticancer drugs such as geldamycin (Wu et al. [2012]). In this study, we explored the macrolide epothilone A, as an aglycon substrate and different nucleotide diphosphate sugars (NDP-D/L-sugars) as sugar donors to generate novel epothilone A glycoside derivatives.

## Materials and methods

### Chemicals and reagents

Epothilone A was available in the laboratory (Samyang Genex Co., Korea). UDP-D-glucose and UDP-D-galactose were purchased from Sigma-Aldrich Chemical Co. (St. Louis, MO, USA). TDP-D-2-deoxyglucose, TDP-L-rhamnose, and GDP-L-fucose were obtained from GeneChem (Daejeon, South Korea). High performance liquid chromatography (HPLC) grade methanol and water were purchased from Mallinckrodt Baker (Phillipsburg, NJ, USA). The remaining chemicals were high-grade products purchased from commercially available sources.

### Plasmid, microorganisms, and culture conditions

A previously constructed pET28 (a)-YjiC (Pandey et al. [2013a]) plasmid was used for transformation in the *E. coli* BL21 (DE3) (Stratagene, La Jolla, CA, USA) expression host for protein production. *E. coli* BL21 (DE3) harboring the pET28 (a)-YjiC strain was grown in Luria- Bertani (LB) liquid medium. The culture was incubated at 37°C with kanamycin (50 μg/mL) supplementation when required.

### Expression and purification of glycosyltransferase

A single transformant of *E. coli* BL21(DE3) harboring pET28 (a)-YjiC was cultured overnight in a shaking incubator in 37°C and 150 rpm. 500 μL of seed culture was transferred to fresh 50 mL LB medium supplemented with 50 μg/mL kanamycin. Once the optical density at 600 nm reached 0.6, the culture was induced with a 0.8 mM final concentration of isopropyl-*β*-D-thiogalactopyranoside and incubated for 20 h at 20°C in shaking incubator at 150 rpm. The cell pellets were harvested via centrifugation at 3,000 rpm for 15 min and washed (vortex followed by centrifugation) with buffer (50 mM Tris–HCl and 10% glycerol of pH 7.4) two times and re-suspended in 1 mL of the same buffer. The cells were sonicated, and the clear lysate was collected by high speed centrifugation at 12,000 rpm for 30 min at 4°C. The clear lysate thus obtained was packed into a His-TALON metal nickel affinity resin (Takara Bio, Shiga, Japan) for 30 min at 4°C. The resin-bound protein was eluted step by step by gravity flow with increasing concentrations of imidazole (10 mM, 100 mM, and 200 mM in buffer containing 200 mM NaCl and 20 mM Tris–HCl, pH 7.4). Fractions containing purified protein were analyzed by 12% sodium dodecyl sulfate polyacrylamide gel electrophoresis (SDS-PAGE) and further concentrated using Amicon Ultra-15 filters (Millipore, 30 K NMWL device; Milford, MA, USA). The purified and concentrated protein was stored in buffer containing 50 mM Tris–HCl, pH 7.4, and 10% glycerol at −20°C.

#### In vitro reaction conditions

The glycosylation reaction was carried out under two conditions using purified protein (YjiC) with the epothilone A and UDP-D-glucose, UDP-D-galactose, TDP-D-2-deoxyglucose, TDP-L-rhamnose, and GDP-L-fucose as sugar donors. A lab-scale reaction was conducted using a 100 μL reaction volume and maintaining a final concentration of 50 mM Tris–HCl buffer at pH 7.4, 10 mM MgCl_2_ 6H_2_O, 2 mM substrate (dissolved in Dimethyl sulfoxide (DMSO)) and 4 mM NDP-sugars individually with 30 μg/mL of appropriately diluted enzyme, and the remaining volume was Milli-Q distilled water. The mixture was incubated at 37°C for 3 h and quenched by adding 300 μL chilled methanol (HPLC grade). The reaction mixture without enzyme was kept as a control. The reaction mixtures were centrifuged at 12,000 rpm for 20 min to remove protein precipitates. Thus, the mixture was finally analyzed by UPLC-PDA and HR-QTOF ESI-MS analyses.

The preparative-scale reaction was carried out in a 5 mL volume with purified YjiC (300 μg/mL), 8 mM UDP-D-glucose (~49 mg), 5 mM substrate (~12.3 mg, dissolved in DMSO), 50 mM Tris–HCl (pH 7.4) buffer, and 10 mM MgCl_2_ and incubated for 18 h at 37°C. The reaction was stopped by adding a triple volume of chilled methanol. The reaction mixture was continuously mixed by vortex followed by centrifugation at 12,000 rpm for 30 min at 4°C to remove the denatured protein. The supernatant was concentrated by evaporation and lyophilization.

### Analytical procedures

The reaction mixture was analyzed by reverse-phase UPLC-PDA with a C18 column (ACQUITY UPLC® BEH, C_18_, 1.7 μm) connected to a PDA (UPLC LG 500 nm) at a UV absorbance of 249 nm. The binary mobile phases were composed of solvent A (HPLC grade water) and solvent B (100% methanol, MeOH). Total flow rate was maintained at 0.4 μL/min for the 10 min program. Flow of B (MeOH) was 0% to 5 min and increased to 100% until 8 min followed by constant flow of 0% at 8–10 min and then flow was stopped at 10 min. The HR-QTOF ESI-MS analysis was performed in positive ion mode on an ACQUITY (UPLC, Waters Corp., Billerica, MA, USA) column coupled with SYNAPT G2-S (Water Corp).

The dried preparative scale reaction product was finally purified by preparative HPLC (Shimadzu, Tokyo, Japan) with a C_18_ column (YMC-Pack ODS-AQ (150 × 20 mm I.D., 10 μm) connected to a UV detector at a UV absorbance of 249 nm using a 46 min binary program with flow of solvent B (100% MeOH) and A (HPLC grade water). Flow of B was initially maintained at 20% and increased to 75% until 25 min, 90% remained (25–35 min) and then flow was decreased to 50% at 40 min followed by 20% at 45 min and finally stopped at 46 min with a flow rate of 10 ml/min.

The product was dried, lyophilized, dissolved in methanol-*d*_4_, and subjected to 900 MHz Bruker, BioSpin nuclear magnetic resonance (NMR) analysis including one-dimensional ^1^H NMR, ^13^C NMR, and two-dimensional NMR-correlation Spectroscopy (COSY), rotating-frame nuclear Overhauser effect spectroscopy (ROESY), heteronuclear single quantum coherence (HSQC), and heteronuclear multiple bond connectivity (HMBC) when appropriate.

## Results

### Expression and purification of glycosyltransferase

The recombinant enzyme YjiC (GenBank accession number AAU40842) was heterologously expressed in *E. coli* BL21 (DE3) soluble fraction as an *N*-terminal hexahistidine-tagged fusion protein. The crude protein was purified using nickel affinity chromatography. SDS-PAGE analysis of the protein produced a distinct band corresponding to hexahistidine-tagged YjiC as in our previous report (Pandey et al. [2013a,b]). The purified protein was used for further glycosylation experiments.

### Enzymatic synthesis of the epothilone A glucoside

**Figure 2:**
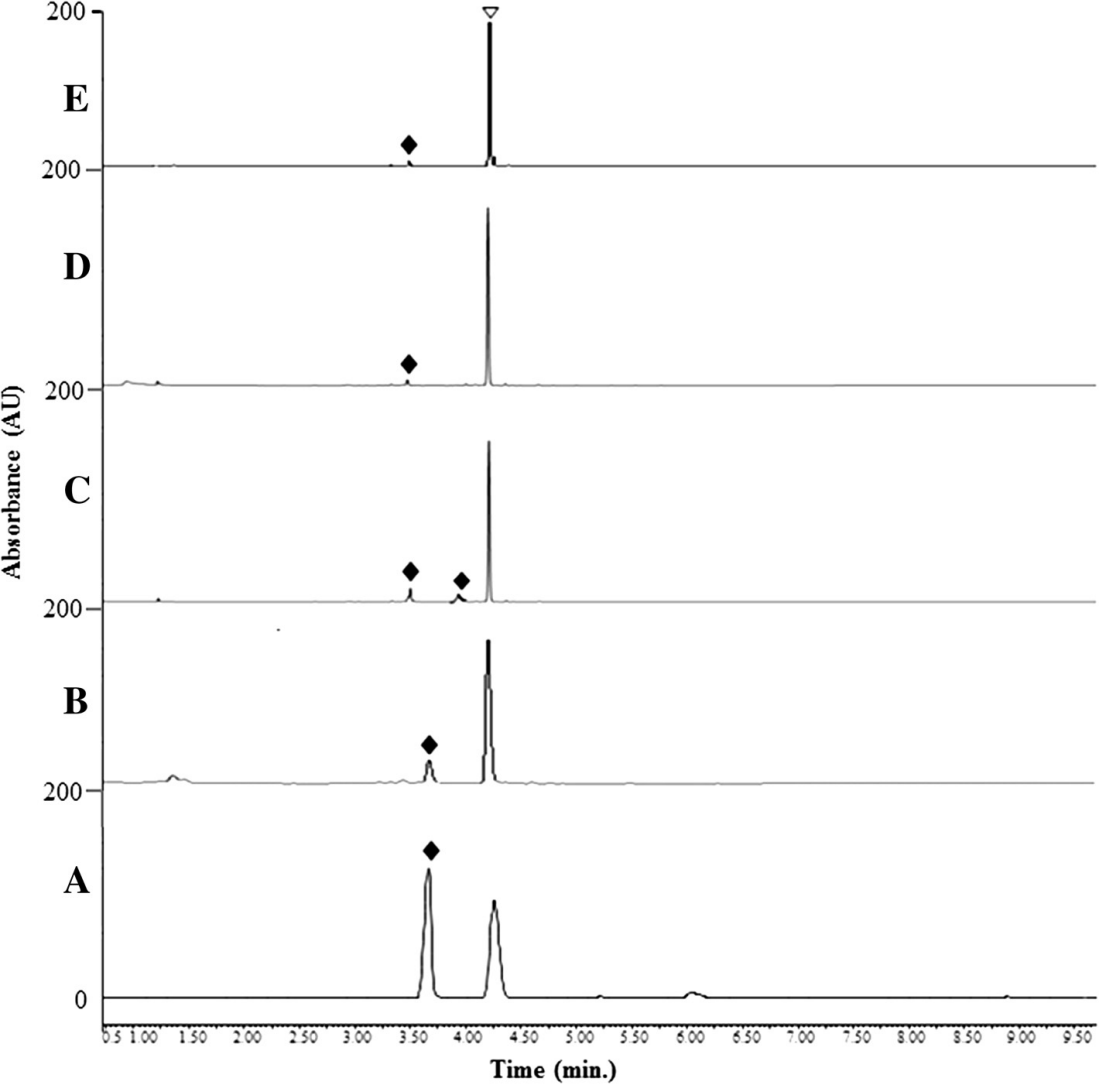
**UPLC-PDA analyses of epothilone A reaction mixture with different sugar donors revealed the new peak(s) generating their respective glycosides as novel compounds.** The chromatogram **(A)** represents the reaction mixture with UDP-D-glucose; **(B)** TDP-D-2-deoxyglucose; **(C)** UDP-D-galactose, **(D)** GDP-L-fucose, and **(E)** and TDP-L-rhamnose with epothilone A. Reaction mixture with UDP-D-galactose generated two novel peaks and has been identified as mono-galactoside and di-galactoside of epothilone A. The triangle shape symbol represents epothilone A aglycon standard whereas the filled diamond shape symbols represent the peaks of rare sugars conjugated epothilone A glycosides.

**Figure 3:**
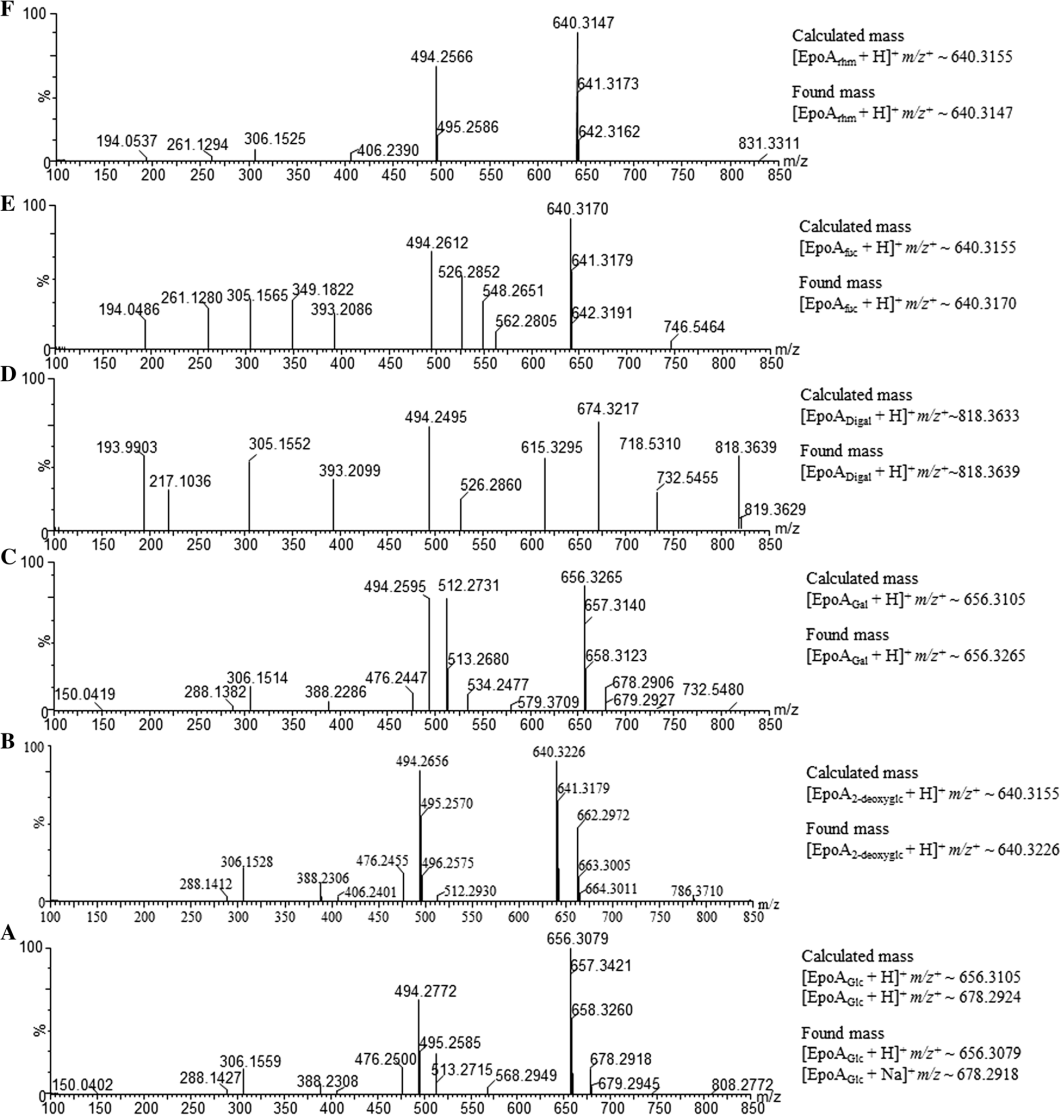
**The UPLC-PDA coupled with HR-QTOF ESI-MS analysis.** Different epothilone A glycosides presenting their respective mass in reference to the aglycon mass [EpoA + H]^+^*m/z*^+^ ~ 494.2576 are as follows: **(A)** Epothilone A 7-*O*-*β*-D-glucoside [EpoA_Glc_ + H]^+^ m/z^+^ ~ 656.3079, **(B)** Epothilone A 7-*O*-*β*-D-2-deoxyglucoside [EpoA_2-deoxyglc_ + H]^+^*m/z*^+^ ~ 640.3226, **(C)** Epothilone A 7-*O*-*β*-D-galactoside [EpoA_Gal_ + H]^+^*m/z*^+^ ~ 656.3265, **(D)** Epothilone A 3, 7-*O*-*β*-D-digalactoside [EpoA_Digal_ + H]^+^*m/z*^+^ ~ 818.3639, **(E)**, Epothilone A 7-*O*-*β*-D-fucoside [EpoA_fuc_ + H]^+^*m/z*^+^ ~ 640.3170 and **(F)** Epothilone A 7-*O*-*β*-D-rhamnoside [EpoA_rhm_ + H]^+^*m/z*^+^ ~ 640.3147.

The purified YjiC enzyme was incubated with epothilone A and UDP-D-glucose in a lab-scale reaction mixture at 37°C for 3 h. The UPLC-PDA chromatogram analysis of the reaction mixture showed a novel peak at a retention time (*t*_*R*_) of 3.51 min (Figure 2(A)). The novel peak was further analyzed by UPLC-PDA coupled with HR-QTOF ESI-MS. The mass analysis identified epothilone A glucoside in the reaction mixture ([EpoA_Glu_ + H]^+^*m/z*^+^: calculated exact mass 656.3105 for C_32_H_50_NO_11_S, found 656.3079, Figure 3(A)). A preparative scale reaction was carried out to further elucidate the structure of the epothilone A glucoside. The preparative scale reaction containing UDP-D-glucose (11 mM, ~49 mg) and epothilone A (5 mM, ~12.3 mg, dissolved in DMSO) resulted in the production of (~1.3 mM, ~4.26 mg) of epothilone A glucoside representing a ~26 % conversion of epothilone A in the single reaction mixture. The reaction product was purified by prep-HPLC, and the dried product was characterized using various NMR studies. Epothilone A had two reactive hydroxyl positions at C-3 and C-7. The YjiC glycosyltransferase transfers the sugar moiety to any of the two hydroxyl groups available. Our previous studies have shown that YjiC transfers the sugar moiety non-regiospecifically to different available hydroxyl positions of flavonoids (Pandey et al. [2013a], b). Thus, the sugar could be coupled with either of the C-3 or C-7 positions of epothilone A.

### Structural elucidation of epothilone A glucoside

**Figure 4:**
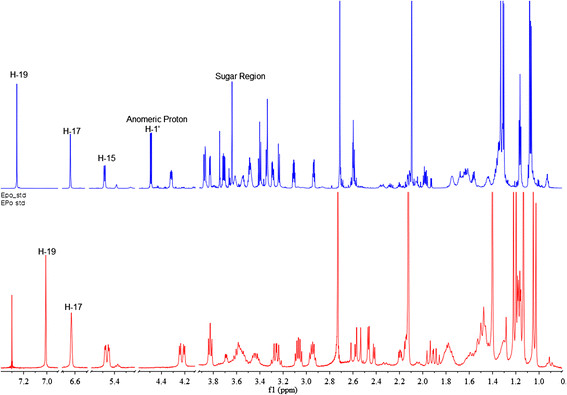
^**1**^**H NMR comparison of epothilone A 7-*O*-*β*-D-glucoside and epothilone A standard.** The NMR analysis shows anomeric proton position and sugar region of the glucoside derivative of epothilone A.

One-dimensional NMR (1D-NMR, ^1^H NMR) and two-dimensional NMR (2D-NMR, ^1^H- ^1^H COSY, ROESY, HMBC, and HSQC) analyses were carried out to elucidate the structure of epothilone A glucoside. The ^1^H NMR study of the purified reaction product showed the presence of an anomeric proton at the chemical shift *δ* = 4.49 ppm (d, *J* = 7.8 Hz, 1H, H-1'), representing an anomeric proton with a beta (*β*) configuration of the sugar moiety, whereas other spectra were observed in the sugar region between *δ* = (3.0–4.0) ppm (Figure 4). Other protons of the epothilone A standard and their products matched exactly when compared with the ^1^H NMR of epothilone as shown in Table 1, Additional file 1: Figure S1 and S2. 2D-NMR analyses were carried out to further confirm the glucosylation position. The ^1^H–^1^H COSY NMR analysis (Additional file 1: Figure S2 (B)) showed that the anomeric proton (*δ* = 4.49 ppm) was in a close relationship with *δ* = 3.2 ppm, which was annotated to H-6 of epothilone by comparing our data with ^1^H NMR of previously identified epothilone A and pentoside of epothilone as epothiloneoside A by Zhao et al. [2010]. Different hydroxylated and epoxidated epothilone A have been produced by microbial biotransformation using *Aspergillus niger* (Wang et al. [2009]) which were characterized by different NMR analyses. Similarly, the ROESY-NMR analysis showed a correlation between the anomeric proton (H-1') and H-24 (*δ* = 1.30), H-6 (*δ* = 3.29), H-2' (*δ* = 3.41), and H-7 (*δ* = 3.83) providing a clue regarding the attachment of a sugar in the C-7 hydroxyl group of epothilone A (Additional file 1: Figure S2 (D)). These results were further supported by HSQC and HMBC analyses showing the correlation of protons with carbons. The HSQC analysis (Additional file 1: Figure S2 (F)) revealed a correlation between H-1' and two anomeric carbons of the sugar moiety (C-1') *δ* = 103 ppm and C-7 of epothilone (*δ* = 74.48) distinctly. Similarly, the HMBC analysis (Additional file 1: Figure S2 (H)) showed a close correlation between the anomeric carbon (C-1', *δ* = 103), H-7 (*δ* = 3.83), and H-6 (*δ* = 3.2), confirming the glucosylation position at the C-7 hydroxyl group. The NMR data were further supported by HR-QTOF ESI-MS/MS analysis in which the fragment of glucose conjugated at C-7 hydroxyl position was observed, but we could not able to find the fragment of epothilone A conjugated at C-3 hydroxyl position (Additional file 1: Figure S3A). Thus, the product was elucidated as epothilone A 7-*O*-*β*-D-glucoside, which is a novel glucoside derivative of epothilone that has not been reported. Zhao and his group have successfully characterized a pentoside of epothilone A as epothiloneoside A in 2010 from *Sorangium cellulosum* So0157-2 (PCT/CN2008/001946) strain culture, and the glycosylated compound was detected as attachment of sugar in C-3 hydroxyl position with a molar mass of 625.29. The compound was determined to be 3-*O*-*α*-D-ribofuranosyl epothilone A (Zhao et al. [2010]) beside this no other reports are available about glycosylated analogues of epothilone A.

**Table 1:** **Comparison of**^**1**^**H NMR of epothilone A standard with epothilone A 7-*O*-*β*-D- glucoside**

**No.**	^**1**^**H NMR of epothilone A standard**	^**1**^**H NMR of epothilone A 7-*O*-*β*-D glucoside**	**No.**	^**1**^**H NMR of epothilone A standard**	^**1**^**H NMR of epothilone A 7-*O*-*β*-D glucoside**
2a	2.58 (dd, 14.5, 10.4, 1-H)	2.60 (d, 4.5, 1-H)	15	5.46 (dd, 8.9, 2.6, 1-H)	5.47 (ddd, 8.5, 2.6, 1.1, 1-H)
2b	2.44 (dd, 14.4, 3.3, 1-H)	2.40-2.24 (m, 1-H)	17	6.63 (dd, 1.8, 1.0, 1-H)	6.63 (dd, 2.1, 1.1, 1-H)
3	4.22 (dd, 10.4, 3.3, 1-H)	4.31 (dd, 9.1, 4.9)	19	7.01 (s, 1-H)	7.26 (s, 1-H)
4	No proton	No proton	21	2.73 (s, 3-H)	2.71 (s, 2-H)
6	3.26 (qd, 6.8, 4.7, 1-H)	3.29 (ddd, 9.7, 5.5, 2.5, 1-H)	22	1.13 (s, 3-H)	1.16 (t, 6.4, 3-H)
7	3.83 (t, 4.4, 1-H)	3.83 (dd, 5.7, 2.1, 1-H)	23	1.40 (s, 3-H)	1.33 (s, H-23)
8	1.84-1.70 (m, 2-H)	1.67-1.60 (m, 2-H)	24	1.21 (d, 6.9, 3-H)	1.30 (d, 6.9, 3-H)
9	1.53-1.42 (m, 2-H)	1.48-1.34 (m, 4-H)	25	1.04 (d, 7.0, 3-H)	1.07 (d, 6.9, 3-H)
10a	1.66-1.52 (m, 1-H)	1.56 (dtd, 12.0, 7.6, 4.5, 1H)	27	2.12 (d, 1.3, 3-H)	2.09 (d, 1.3, 3-H)
10b	1.53-1.42 (m, 1-H)	1.48-1.34 (m, 4-H)	1'	No proton	4.49 (d, 7.8, 1-H)
11a	1.84-1.70 (m, 2-H)	1.67-1.60 (m, 2-H)	2'	No proton	3.41 (t, 9.0, 1-H)
11b	1.53-1.42 (m, 1-H)	1.48-1.34 (m, 4-H)	3'	No proton	3.52-3.47 (m, 2-H)
12	2.95 (dt, 7.4, 3.9, 1-H)	2.94 (dt, 8.1, 4.1, 1-H)	4'	No proton	3.38-3.34 (m, 1-H)
13	3.07 (dt, 8.2, 4.2, 1-H)	3.11 (dt, 8.4, 4.4, 1-H)	5'	No proton	3.52-3.47 (m,2-H)
14a	2.24-2.11 (m, 1-H)	2.12 (ddd, 15.0, 4.6, 2.7, 1-H)	6a'	No proton	3.88 (dd, 11.8, 2.4, 1-H)
14b	2.00-1.82 (m, 1-H)	1.98 (dt, 14.8, 8.2, 1-H)	6b'	No proton	3.71 (dd, 11.8, 5.5, 1-H)

^1^H NMR of epothilone A standard was performed at 300 MHz in CDCl_3_ whereas the ^1^H NMR of epothilone A glucoside was determined at 900 MHz in CD_3_OD. Multiplicities are indicated by s (singlet), d (doublet), t (triplet), q (quartet), qn (quintet), m (multiplet) and br (broad) if necessary. Chemical shifts are reported in parts per million (ppm) and coupling constants *J* are given in Hz when appropriate.

### Time-dependent study of the glucosylation reaction

**Figure 5:**
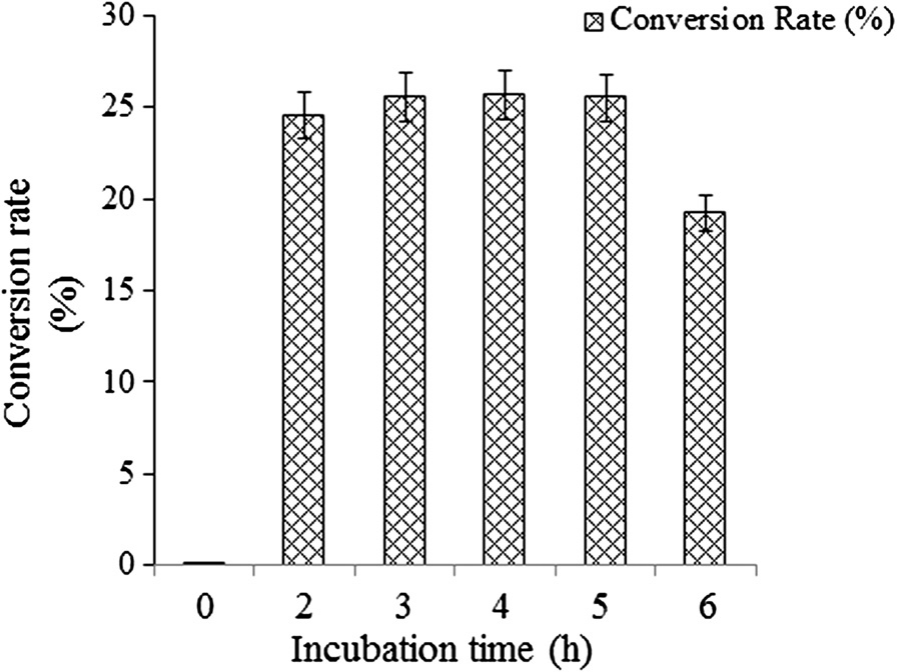
**The time dependent study of epothilone A glucosylation.** The enzymatic conversion of epothilone A to epothilone A 7-*O*-*β*-D-glucoside was determined at different time intervals. The *error bars* represent the standard deviation of three independent experiments.

A glycosylation reaction with epothilone A and UDP-D-glucose was conducted as described above to identify the incubation time needed for maximum conversion of epothilone A to epothilone A 7-*O*-*β*-D-glucoside and to find out the stability of thus produced glucoside derivative in the reaction mixture. Samples were taken out at different times (0 h, 2 h, 4 h, 5 h, 7 h, and 8 h) and were subjected to UPLC-PDA at an absorbance of 249 nm. Under the 8 h reaction mixture analyses, the maximum glucoside conversion was achieved between 3 h to 5 h of incubation (25.55–25.56%) at 37°C, whereas product formation declined after 5 h (Figure 5). We did not achieve a significantly higher conversion rate after extending the incubation period. Instead, the product was degraded when the incubation time was increased. Two possible reasons explain the decreasing concentration of epothilone A 7-*O*-*β*-D-glucoside in the reaction mixture with a longer incubation. The first could be deglycosylation of epothilone A 7-*O*-*β*-D-glucoside to epothilone A and UDP-D-glucose, as this property of YjiC has been previously characterized in our study. A second reason could be the thermodynamic instability of the compound in the reaction mixture.

### Enzymatic synthesis of diverse epothilone A glycosides

**Figure 6:**
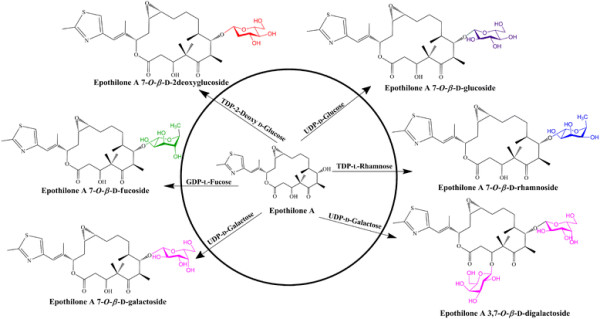
**Glycosylation reaction of epothilone A catalyzed by the YjiC enzyme with diverse NDP-D/L-sugars.** Epothilone A is coupled with five different sugar moeities (glucose, galactose, 2-deoxyglucose, rhamnose, and fucose) to generate six diverse epothilone A glycoside analogues. All the identified epothilone A glycosides are novel compounds.

After confirming production of epothilone A 7-*O*-*β*-D-glucoside, we attempted enzymatic glycosylation of epothilone A using various NDP-D/L-sugars. Most of the NDP-sugars used for the reaction were rare sugars. Thus, identical reactions were carried out varying only the NDP-sugar donor at 37°C. UPLC-PDA coupled with HR-QTOF ESI-MS/MS analyses (Figures 2 and 3) revealed the presence of glycosylated products in all of the independent reactions of YjiC with epothilone A and UDP-D-galactose, TDP-D-2-deoxyglucose, GDP-L-fucose, and TDP-L-rhamnose. Because of the lack of excess availability of NDP-D/L-sugars, their long enzymatic synthesis process and very low conversion rate of epothilone A to respective glycosides (Figure 2), we were unable to characterize those products by NMR analysis. Because the structurally elucidated epothilone A glucoside showed that the C-7 hydroxyl position of epothilone A is prominent for glycosylation by YjiC, other NDP-D/L-sugar reaction mixtures analyzed by high resolution exact mass spectra (Figure 3) confirmed the products as epothilone A 7-*O*-*β*-D-galactoside (*t*_*R*_:4.15 min, [EpoA_Gal_ + H]^+^*m/z*^+^: calculated exact mass 656.3105 for C_32_H_50_NO_11_S, found 656.3265), epothilone A 3,7-*O*-*β*-D-digalactoside (*t*_*R*_:3.78 min, [EpoA_Digal_ + H]^+^*m/z*^+^: calculated exact mass 818.3633 for C_38_H_60_NO_16_S, found 818.3639) (Figure 3(C and D)), epothilone A 7-*O*-*β*-D-2-deoxyglucoside (*t*_*R*_:3.46 min, [EpoA_2-deoxyglc_ + H]^+^*m/z*^+^: calculated exact mass 640.3155 for C_32_H_50_NO_10_S, found 640.3226) (Figure 3(B)), epothilone A 7-*O*-*β*-L-fucoside (*t*_*R*_:4.72 min, [EpoA_fuc_ + H]^+^*m/z*^+^: calculated exact mass 640.3155 for C_32_H_50_NO_10_S, found 640.3170) (Figure 3(E)), and epothilone A 7-*O*-*β*-L-rhamnoside (*t*_*R*_:3.67 min, [EpoA_rhm_ + H]^+^*m/z*^+^: calculated exact mass 640.3155 for C_32_H_50_NO_10_S, found 640.3147) (Figure 3(F)). Thus produced epothilone glycosides and their position of glycosylation were also further confirmed by HR-QTOF ESI-MS/MS analyses (Additional file 1: Figure S3 (B-E)). The MS/MS fragmentation of each epothilone A glycosides showed the presence of prominent fragment of epothilone A with respective sugars attached at C-7 hydroxyl position. The fragment of sugar attached at the C-3 hydroxyl position was not observed in ESI-MS/MS spectra of all glycoside derivatives. The structural elucidation of epothilone A 7-*O*-*β*-D-glucoside by NMR studies and HR-QTOF ESI-MS/MS analysis of all epothilone A glycosides proved that YjiC regioselectively glycosylate at 7-hydroxyl position of epothilone A. Although all of the produced epothilone A glycosides were monoglycosides, a digalactoside derivative (epothilone A 3, 7-*O*-*β*-D-digalactoside) was also produced using UDP-D-galactose as the sugar donor (Figure 6).

## Discussion

Interest in epothilones immediately soared when they were tested and found to have extraordinary inhibitory effects on cell proliferation and cell death in paclitaxel-resistant tumor cell lines at up to 5,000-fold lower concentrations than that of taxol (Carlomagno et al. [2003]). Drugs that target microtubules are the most commonly prescribed anticancer therapeutics in current clinical practice. Epothilones bear similar potency and were classified as a new class of antimicrotubule agents that share the equipotent structural and functional properties with taxanes among which few have already passed preclinical studies as cancer therapeutic agents (Zhao et al. [2010]; Goodin et al. [2004]). Ixabepilone, a semisynthetic analog of epothilone B, was approved by the US Food and Drug Administration in October 2007 to treat metastatic or locally advanced breast cancer as monotherapy (Donovan and Vahdat [2008]).

Microtubules play a crucial role in cellular processes such as cell motility, intracellular trafficking, cell division, and cell maintenance. Thus, current compounds targeting microtubules are being developed as cancer chemotherapeutic agents (Bollag et al. [1995]). The microtubule targeting compounds engage at the mitotic spindle checkpoint where they block cell cycle progression at mitosis ultimately leading to apoptosis (Bhat and Setaluri [2007]). Nevertheless, epothilones were originally described as natural product fungicidal macrolides (Hofle and Reichenbach [2005]). However, later studies revealed the microtubule polymerization property at submicromolar concentrations and soon their potency was verified to replace paclitaxel (Goodin et al. [2004]). Epothilone arrests cells at the mitotic G2/M transition phase (a rapid cell growth and protein synthesis period) at low nanomolar concentrations eventually leading to apoptosis during mitosis (Harle and Bechthold [2009]). Since the discovery of such cytotoxic potential bearing microtubule-stabilizing agents, total synthesis has been a challenge to most organic chemists (He et al. [2001]). The reason is an adequate supply of epothilones as healing agents for clinical studies. Similar class of microtubules stabilizing anticancer drugs such as discodermolide (ter Haar et al. [1996]), elutherobin (Long et al. [1998]), sarcodictyins A and B (Hamel et al. [1999]), and laulimalide (Mooberry et al. [1999]) have been reviewed by Karl-Heinz Altmann in [2001].

Taxens are considered to be first-line option for metastatic breast cancer, but their utility was compromised because of resistivity (Cortes et al. [2012]). Thus, epothilone A was selected for in vitro glycosylation in the current study because it has high demand in the cancer therapeutic development possessing high cytotoxic effects in taxane sensitive cell lines including P-glycoprotein overexpression (Cortes et al. [2012]). Cytotoxic effects of epothilones with diverse human cancer cell line including lung cancer cell line (NCI-H460) (Kim et al. [2003]), ovarian cancer cell line (SKOV-3) (Rogalska et al. [2013]), breast cancer cell lines (Cheng et al. [2008]), etc. have been reported in dose dependent manner. However, six different analogues of epothilones are in preclinical and clinical trials including patupilone (epothilone A and B), ixabepilone (BMS247550), BMS 310705, sagopilone (ZK-EPO), KOS-862 (epothilone D), and KOS-1584, etc. where all of these compounds were modified from major product of myxo-bacterium (epothilone A and B) (Cheng et al. [2008]). So, due to low water solubility but high therapeutic value we hereby try to synthesize the glucosyl analogues of epothilone A using in vitro glycosylation approach. Six different glycoside derivatives were successfully produced in the reaction catalyzed by YjiC in the presence of diverse NDP-D/L-sugars as explained in result section. Maximum glucoside conversion rate was determined from the time dependent study of epothilone A coupled with UDP-D-glucose. Approximately 26% glucoside was achieved in between 3 h to 5 h of incubation time at 37°C. As explained in the result section, after 5 h the catalytic activity of YjiC preferred reverse direction while extending the incubation time as the product formation was declining. Another reason is the structural rigidity of the aglycon which results in low substrate availability to the glycosyltransferase (Wu et al. [2012] and Richard et al. [1999]). The exact catalytic activity of the diverse substrate flexible bacterial glycosyltransferase, YjiC has not been determined so far. So this could be one advantage in modification of diverse antibiotics or other natural products with different glycosylation approach. The validated in vitro glycosylation reaction of epothilone A with diverse sugar donors and new putative glucoside peak(s) formation during UPLC-PDA analysis were further verified through high resolution ESI MS/MS analysis revealed the successive generation of six different glycosylated analogues. Epothilone A glucoside was referenced for further (1-D and 2-D) NMR analysis which ultimately exposed the C-7 hydroxyl position sugar conjugated compound as epothilone A 7-*O-β*-D-glucoside. This concludes the C-7 hydroxyl position of epothilone A is regiospecific for the glycosylation because as we can see the C-3 hydroxyl position is much hindered by the presence of two keto-groups in parallel position.

Engineering the sugar moiety in natural products has always generated novel therapeutic drugs and enhanced their parental properties based on structural, functional, and cell recognition (Desmet et al. [2012]). Thus, these modified novel glycosyl analogues of epothilone A could be the new drugs for treating cancers in the future. In the near future, different glycosylated analogues of epothilone A with improved efficacy will be exploited to develop further clinical applications. But additional experimental evidence including biological activities will certainly help to describe the structural and functional efficacy of newly synthesized derivatives. The approach of producing glycodiversified compounds definitely adds significance to the development of novel drugs for treating various diseases including cancer. As the yield of novel compounds is too low at the current stage, engineering of the YjiC glycosyltransferases is essential for improved catalytic efficiency of the enzyme to industrially target epothilone A glycoside derivatives.

## Competing interests

The authors declare that they have no competing interests.

## Additional file

## Supplementary Material

Additional file 1: **1-dimensional 1H-NMR and 13C- NMR of epothilone A standard.** 13C-NMR and 2-dimensional NMR analyses of epothilone A 7-O-beta-D-glucoside. HR-QTOF ESI-MS/MS analysis of diverse Epothilone A glycosides. Click here for file
