# Supplementary material for: Enzymatic synthesis of epothilone A glycosides
Source: AMB Express. 2014 Mar 20;4:31. doi: 10.1186/s13568-014-0031-1 (PMC4052672; doi:10.1186/s13568-014-0031-1)
Supplement: Additional file 1: — 1-dimensional 1H-NMR and 13C- NMR of epothilone A standard. 13C-NMR and 2-dimensional NMR analyses of epothilone A 7-O-beta-D-glucoside. HR-QTOF ESI-MS/MS analysis of diverse Epothilone A glycosides. [file s13568-014-0031-1-S1.pdf]

Supplementary Information for AMB Express

Enzymatic Synthesis of Epothilone A Glycosides

Prakash Parajuli[1†], Ramesh Prasad Pandey[1†], Niranjan Koirala[1], Yeo Joon Yoon[2], Byung-Gee Kim[3], and Jae Kyung Sohng[1]*

[1]*Institute of Biomolecule Reconstruction (iBR), Department of Pharmaceutical Engineering, SunMoon University, Asan-si, Chungnam 336-708, Republic of Korea*

[2]*Department of Chemistry and Nano Science, Ewha Womans University, Seoul 120-750, Republic of Korea*

[3]*Laboratory of Molecular Biotechnology and Biomaterials, School of Chemical and Biological Engineering, Seoul National University, Seoul, Republic of Korea*

[†] **These authors are equally contributed to this work.**

*Corresponding author: Prof. J. K. Sohng

E-mail: sohng@sunmoon.ac.kr

Tel: +82(41)530-2246,

Fax: +82(41)544-2919

Table of contents

Figure S1. 1-dimensional ^{1}H-NMR and ^{13}C- NMR of epothilone A standard

A. ^{1}H NMR

B. ^{13}C NMR

Figure S2. 1-dimensional and 2-dimensional Nuclear magnetic resonance (NMR) studies of epothilone A glucosides.

A. ^{1}H NMR of epothilone A glucoside

B. ^{1}H - ^{1}H COSY NMR

C. ^{1}H - ^{1}H COSY NMR close view of sugar region

D. ROESY

E. ROESY close view of sugar region

F. HSQC

G. HSQC close view of sugar region

H. HMBC

I. HMBC close view of sugar region

Figure S3. HR-QTOF ESI-MS/MS analysis of epothilone A glycosides

Figure S1

A. ^{1}H NMR

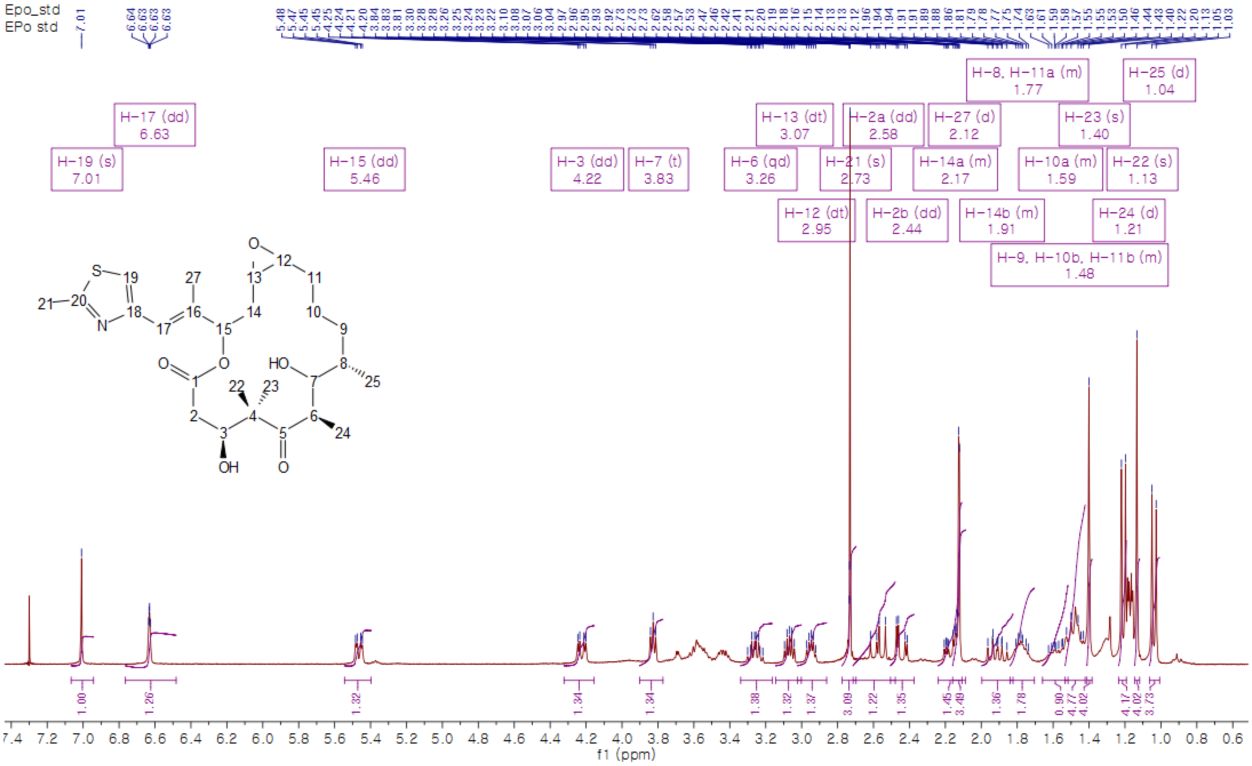

^{1}H NMR (300 MHz, $CDCl_3$) δ 7.01 (s, 1H, H-19), 6.63 (dd, *J* = 1.8, 1.0 Hz, 1H, H-17), 5.46 (dd, *J* = 8.9, 2.6 Hz, 1H, H-15), 4.22 (dd, *J* = 10.4, 3.3 Hz, 1H, H-3), 3.83 (t, *J* = 4.4 Hz, 1H, H-7), 3.26 (qd, *J* = 6.8, 4.7 Hz, 1H, H-6), 3.07 (dt, *J* = 8.2, 4.2 Hz, 1H, H-13), 2.95 (dt, *J* = 7.4, 3.9 Hz, 1H, H-12), 2.73 (s, 3H, H-21), 2.58 (dd, *J* = 14.5, 10.4 Hz, 1H, H-2a), 2.44 (dd, *J* = 14.4, 3.3 Hz, 1H, H-2b), 2.24 – 2.11 (m, 1H, H-14a), 2.12 (d, *J* = 1.3 Hz, 3H, H-27), 2.00 – 1.82 (m, 1H, H-14b), 1.84 – 1.70 (m, 2H, H-8, H-11a), 1.66 – 1.52 (m, 1H, H-10a), 1.53 – 1.42 (m, 4H, H-9, H-10b, H-11b), 1.40 (s, 3H, H-23), 1.21 (d, *J* = 6.9 Hz, 3H, H-24), 1.13 (s, 3H, H-22), 1.04 (d, *J* = 7.0 Hz, 3H, H-25).

B. ^{13}C NMR

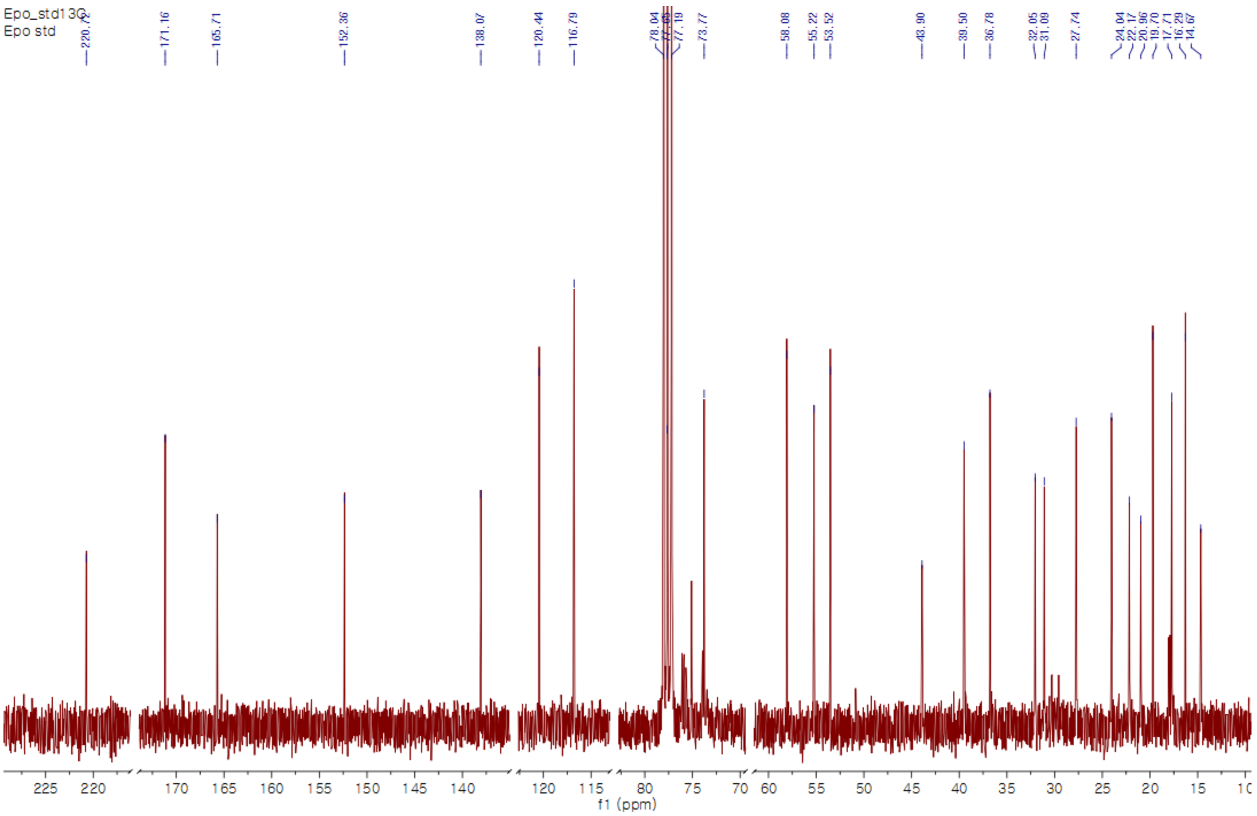

^{13}C NMR (75 MHz, $CDCl_3$) δ 14.674, 16.295, 17.710, 19.699, 20.961, 22.173, 24.036, 27.739, 31.089, 32.047, 36.780, 39.500, 43.899, 53.517, 55.220, 58.084, 73.771, 77.188, 77.649, 78.038, 116.789, 120.443, 138.074, 152.363, 165.708, 171.163, 220.723.

Figure S2

A. ^{1}H NMR

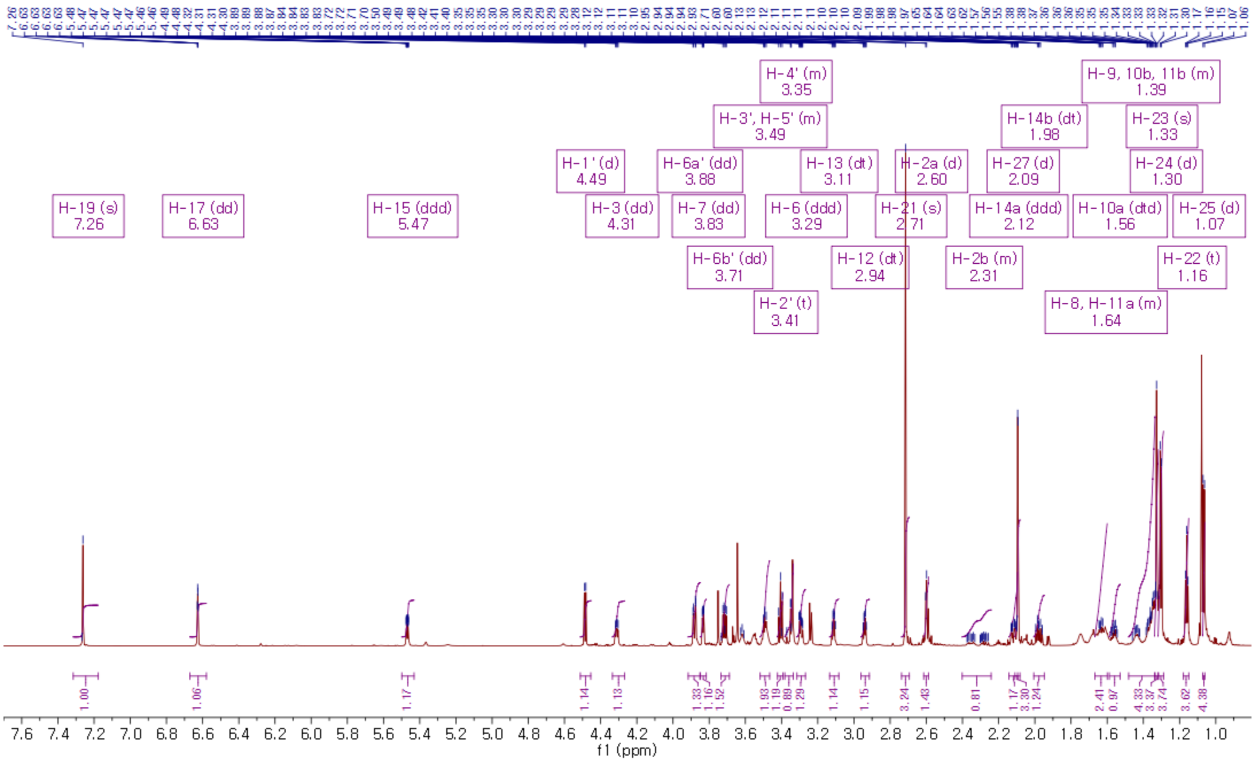

^{1}H NMR (900 MHz, CD_3OD) δ 7.26 (s, 1H, H-19), 6.63 (dd, *J* = 2.1, 1.1 Hz, 1H, H-17), 5.47 (ddd, *J* = 8.5, 2.6, 1.1 Hz, 1H, H-15), 4.49 (d, *J* = 7.8 Hz, 1H, H-1'), 4.31 (dd, *J* = 9.1, 4.9 Hz, 1H, H-3), 3.88 (dd, *J* = 11.8, 2.4 Hz, 1H, H-6a'), 3.83 (dd, *J* = 5.7, 2.1 Hz, 1H, H-7), 3.71 (dd, *J* = 11.8, 5.5 Hz, 1H, H-6b'), 3.52 – 3.47 (m, 2H, H-3', H-5'), 3.41 (t, *J* = 9.0 Hz, 1H, H-2'), 3.38 – 3.34 (m, 1H, H-4'), 3.29 (ddd, *J* = 9.7, 5.5, 2.5 Hz, 1H, H-6), 3.11 (dt, *J* = 8.4, 4.4 Hz, 1H, H-13), 2.94 (dt, *J* = 8.1, 4.1 Hz, 1H, H-12), 2.71 (s, 3H, H-21), 2.60 (d, *J* = 4.5 Hz, 1H, H-2a), 2.40 – 2.24 (m, 1H, H-2b), 2.12 (ddd, *J* = 15.0, 4.6, 2.7 Hz, 1H, H-14a), 2.09 (d, *J* = 1.3 Hz, 3H, H-27), 1.98 (dt, *J* = 14.8, 8.2 Hz, 1H,H-14b), 1.67 – 1.60 (m, 2H, H-8, 11a), 1.56 (dtd, *J* = 12.0, 7.6, 4.5 Hz, 1H,H-10a), 1.48 – 1.34 (m, 4H, H-9, H-10b, 11b), 1.33 (s, 3H, H-23), 1.30 (d, *J* = 6.9 Hz, 3H, H-24), 1.16 (t, *J* = 6.4 Hz, 3H, H-22), 1.07 (d, *J* = 6.9 Hz, 3H, H-25).

B. ^{1}H - ^{1}H COSY NMR (900 MHz, CD_3OD)

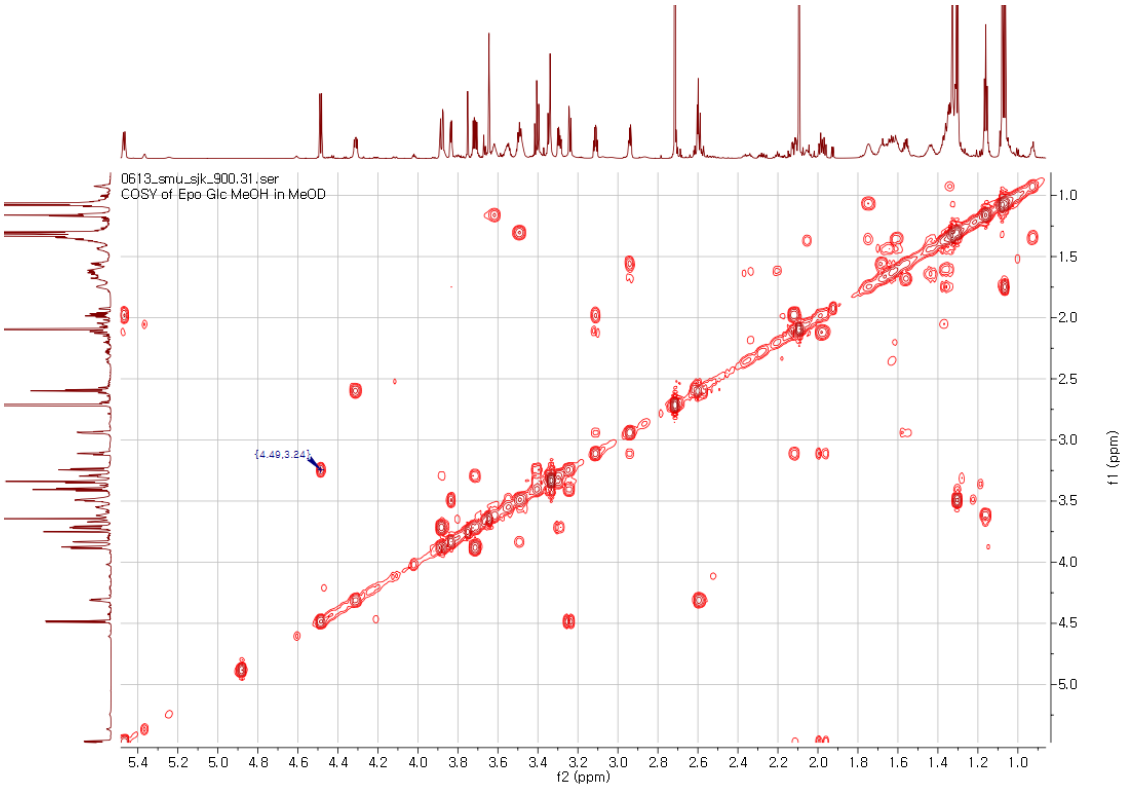

C. ^{1}H - ^{1}H COSY NMR close view of sugar region

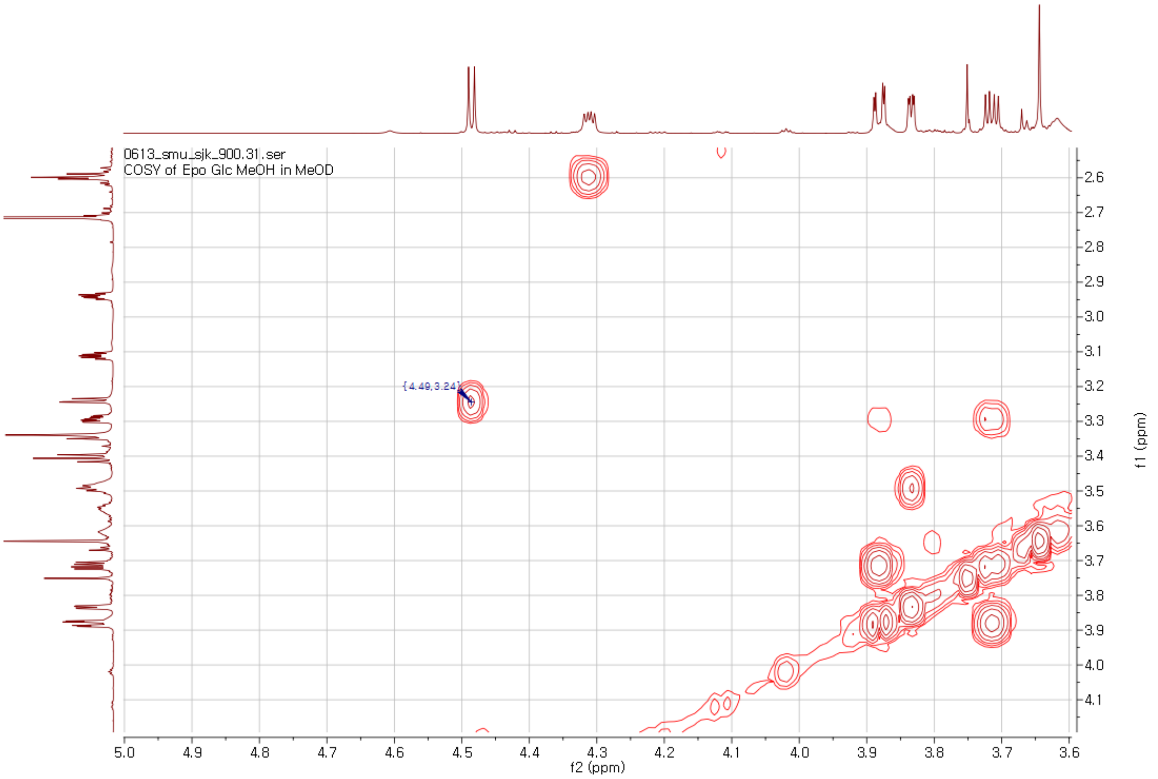

D. ROESY (900 MHz, CD_3OD)

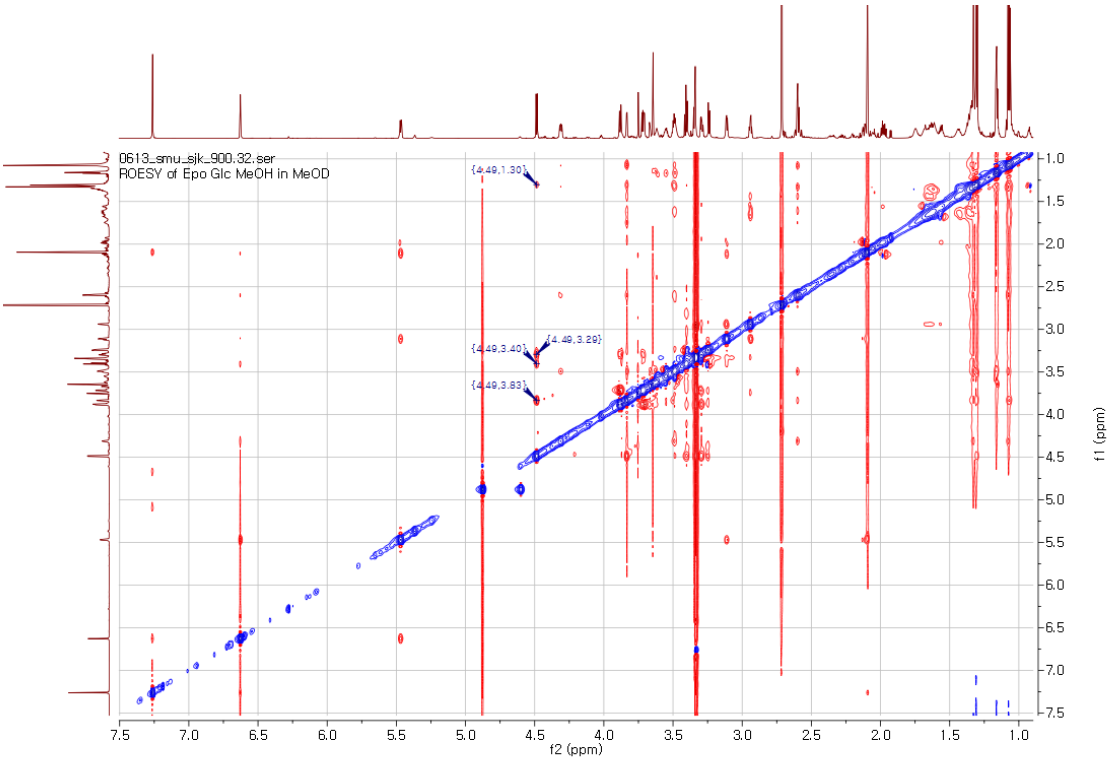

E. ROESY close view of sugar region

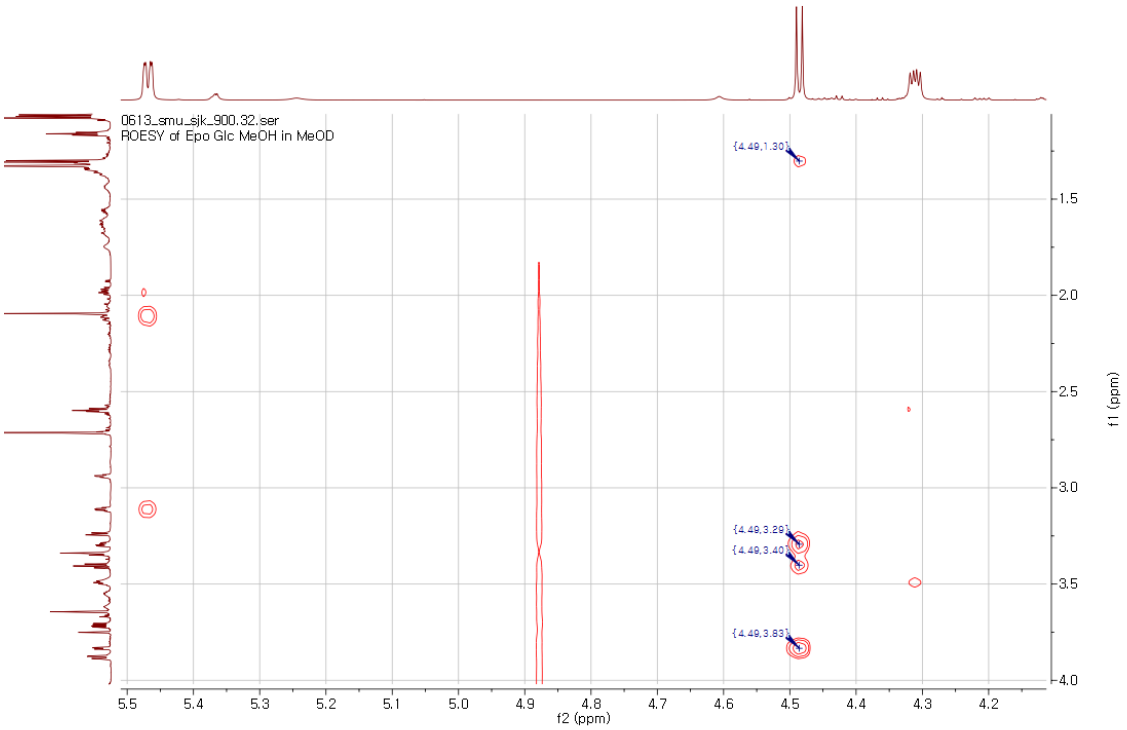

F. HSQC (900 MHz, CD_3OD)

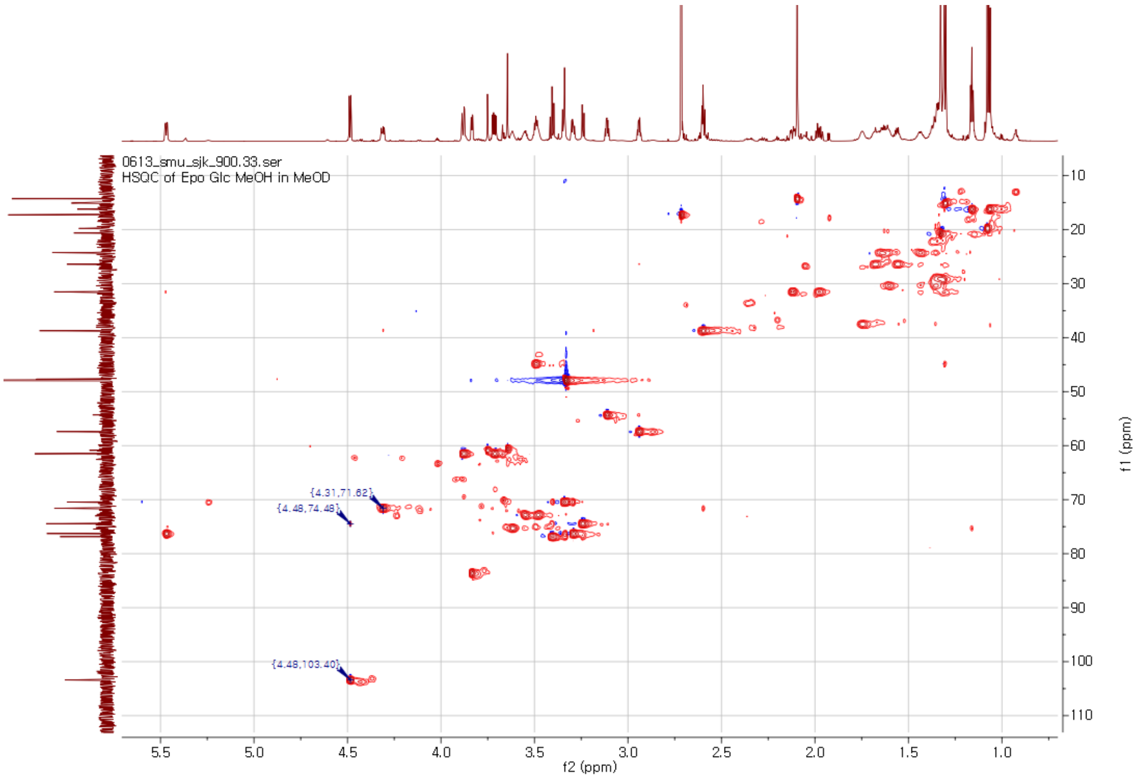

G. HSQC close view of sugar region

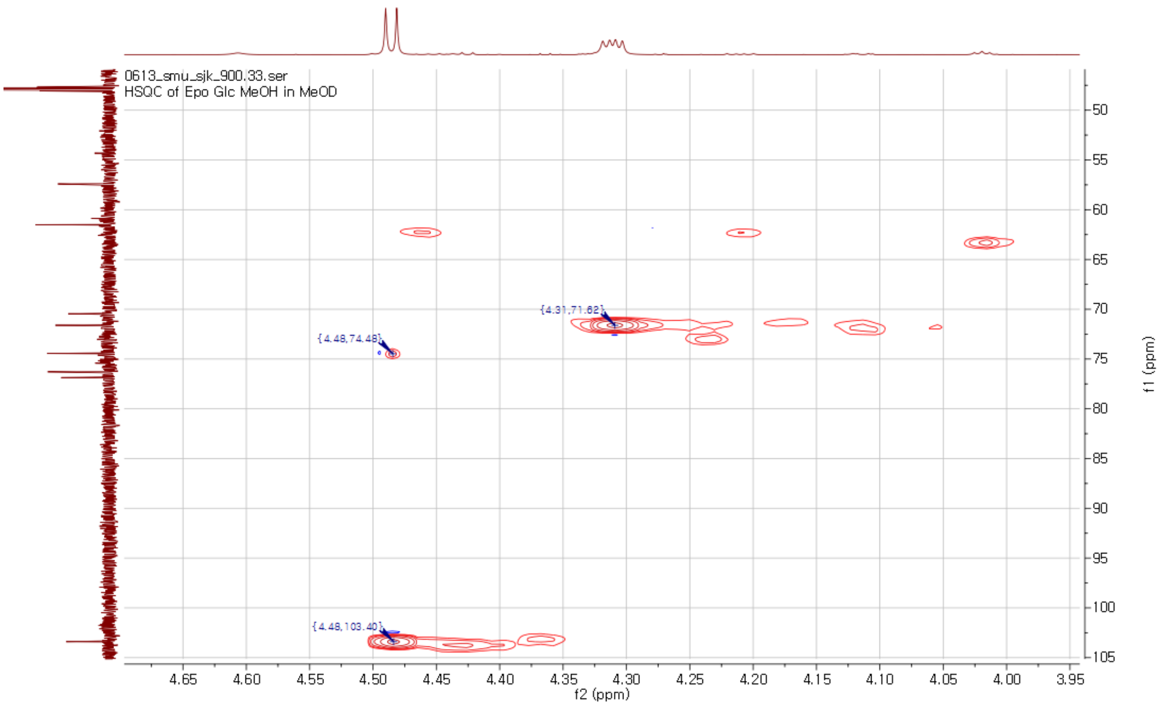

H. HMBC (900 MHz, CD_3OD)

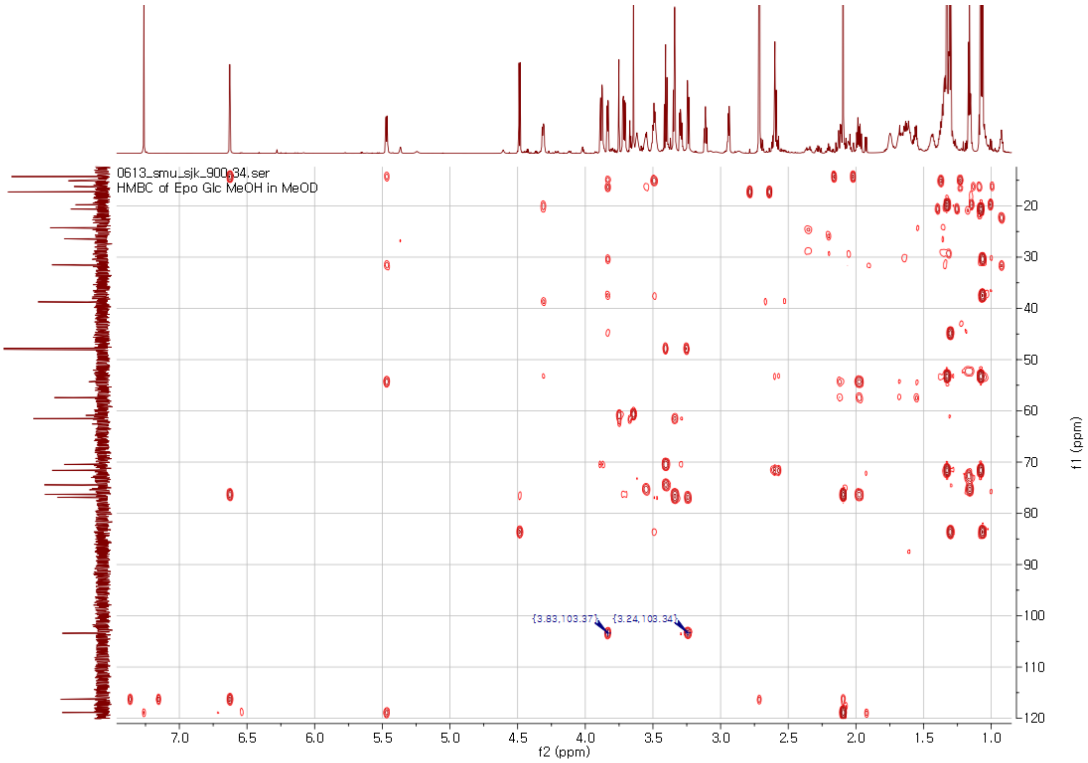

I. HMBC close view of sugar region

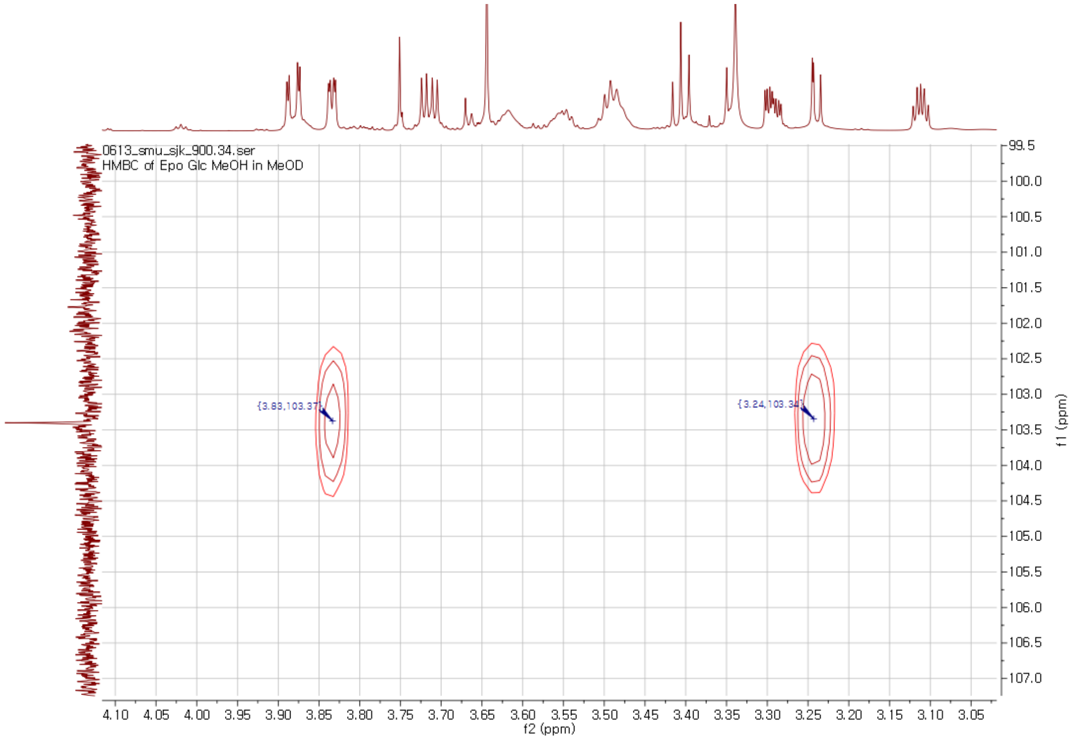

103 **Figure S3.**

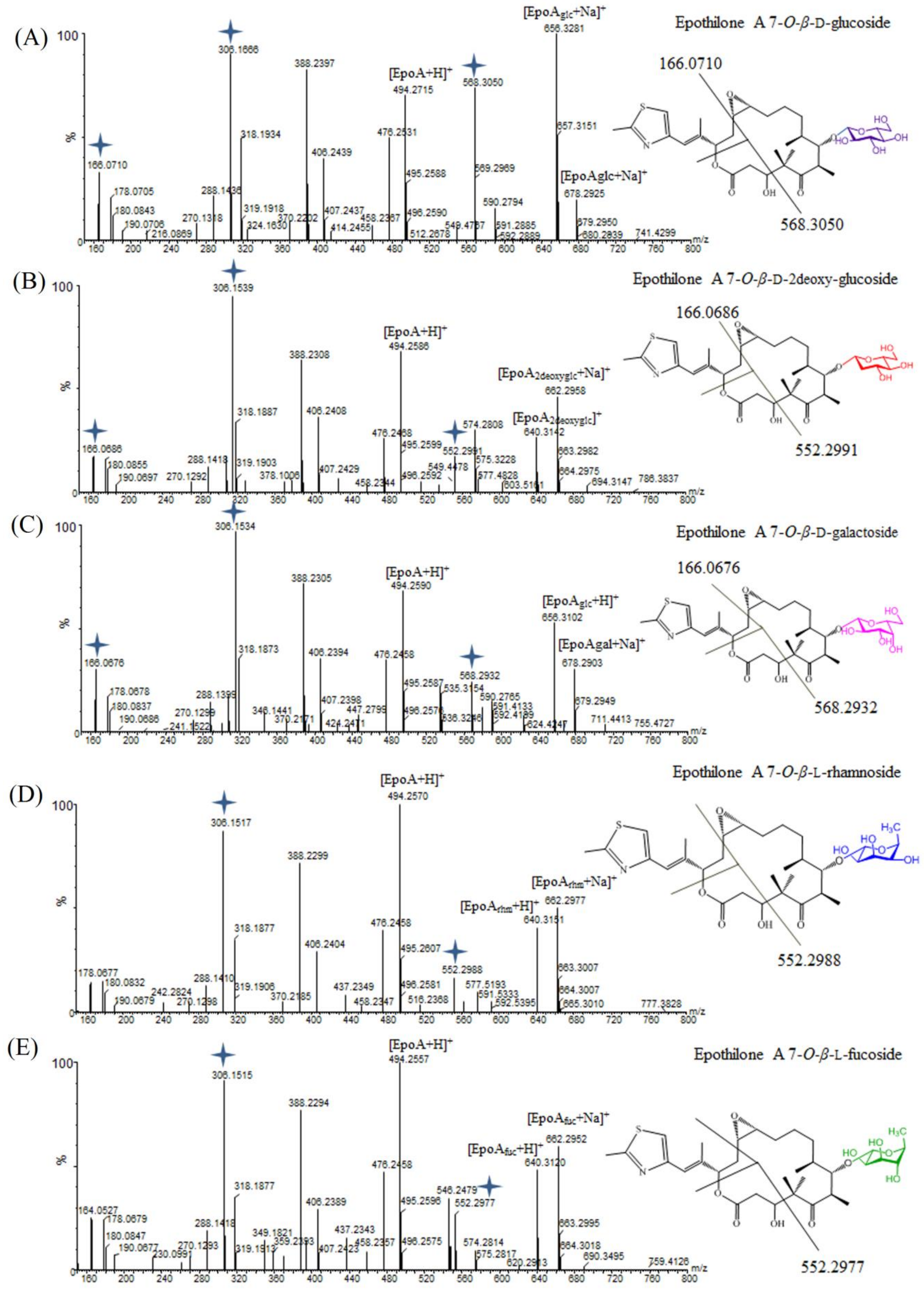

104
